# Enhancing angiogenesis in peri-implant soft tissue with bioactive silk fibroin microgroove coatings on zirconia surfaces

**DOI:** 10.1093/rb/rbae068

**Published:** 2024-06-17

**Authors:** Zhihan Wang, Palati Tuerxun, Takkun Ng, Yinuo Yan, Ke Zhao, Yutao Jian, Xiaoshi Jia

**Affiliations:** Guanghua School of Stomatology, Hospital of Stomatology, Sun Yat-sen University, Guangzhou, Guangdong 510055, China; Guangdong Provincial Key Laboratory of Stomatology, Guangzhou, China; Guanghua School of Stomatology, Hospital of Stomatology, Sun Yat-sen University, Guangzhou, Guangdong 510055, China; Guangdong Provincial Key Laboratory of Stomatology, Guangzhou, China; Guanghua School of Stomatology, Hospital of Stomatology, Sun Yat-sen University, Guangzhou, Guangdong 510055, China; Guangdong Provincial Key Laboratory of Stomatology, Guangzhou, China; Guanghua School of Stomatology, Hospital of Stomatology, Sun Yat-sen University, Guangzhou, Guangdong 510055, China; Guangdong Provincial Key Laboratory of Stomatology, Guangzhou, China; Guanghua School of Stomatology, Hospital of Stomatology, Sun Yat-sen University, Guangzhou, Guangdong 510055, China; Guangdong Provincial Key Laboratory of Stomatology, Guangzhou, China; Guanghua School of Stomatology, Hospital of Stomatology, Sun Yat-sen University, Guangzhou, Guangdong 510055, China; Guangdong Provincial Key Laboratory of Stomatology, Guangzhou, China; Guanghua School of Stomatology, Hospital of Stomatology, Sun Yat-sen University, Guangzhou, Guangdong 510055, China; Guangdong Provincial Key Laboratory of Stomatology, Guangzhou, China

**Keywords:** zirconia surface, silk fibroin, microgrooves coating, angiogenesis, soft tissue

## Abstract

Zirconia abutments and restorations have improved the aesthetic appeal of implant restoration, yet peri-implantitis poses a significant threat to long-term success. The soft tissue surrounding implants is a crucial biological barrier against inflammation and subsequent bone loss. Peri-implantitis, akin to periodontitis, progresses rapidly and causes extensive tissue damage. Variations in tissue structure significantly influence disease progression, particularly the lower vascular density in peri-implant connective tissue, compromising its ability to combat infection and provide essential nutrients. Blood vessels within this tissue are vital for healing, with angiogenesis playing a key role in immune defense and tissue repair. Enhancing peri-implant soft tissue angiogenesis holds promise for tissue integration and inflammation control. Microgroove surfaces have shown potential in guiding vessel growth, but using subtractive technologies to carve microgrooves on zirconia surfaces may compromise mechanical integrity. In this study, we utilized inkjet printing to prepare bioactive silk fibroin microgrooves (SFMG) coating with different sizes on zirconia surfaces. SFMG coating, particularly with 90 µm width and 10 µm depth, effectively directed human umbilical vein endothelial cells (HUVECs) along microgrooves, promoting their proliferation, migration, and tube formation. The expression of vascular endothelial growth factor A and fibroblast growth factor in HUVECs growing on SFMG coating was upregulated. Additionally, the SFMG coating activated the PI3K-AKT pathway and increased glycolytic enzyme gene expression in HUVECs. In conclusion, SFMG coating enhances HUVEC growth and angiogenesis potential by activating the PI3K-AKT pathway and glycolysis, showing promise for improving tissue integration and mitigating inflammation in zirconia abutments and restorations.

## Graphical Abstract

**Figure:**
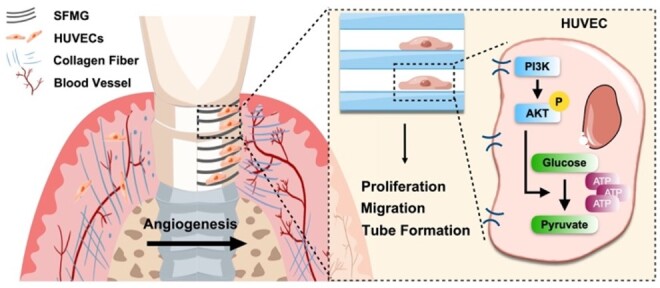

## Introduction

Dental implant restorations have achieved broad acceptance in clinical practice, providing numerous benefits over traditional partial dentures. The effective integration of dental implants with the adjacent tissues is a pivotal factor influencing the success rates of implants. The soft tissue around the abutment forms a ‘cuff-like’ barrier, isolating the bone surrounding the implant from the oral environment. This barrier is crucial for biological healing, preventing inflammation around the implant and ensuring its long-term retention [1]. Compared to titanium-based abutments, zirconia abutments not only offer superior aesthetic properties [2] and inhibit bacterial adhesion [3–5], but also enhance biocompatibility [6], increasing their utility in clinical settings. However, despite these advantages, the biologically inert surface of zirconia can pose a challenge for the integration of the soft tissue surrounding the implant. This lack of biological interaction can make the soft tissue more susceptible to issues that compromise the implant’s stability over time.

Peri-implantitis exhibits a higher incidence and faster progression than periodontitis [7]. The structural characteristics of tissues play a crucial role in the progression of diseases. Connective tissues around implants are essential for stability, creating a biological barrier that isolates the implant from external elements and mitigates potential inflammation [8]. Epithelial tissues surrounding implants resemble those around natural teeth, yet peri-implant connective tissues exhibit significantly fewer vascular structures compared to periodontal connective tissue [9, 10]. Blood supply provides tissues with oxygen, nutrients, various cell types and growth factors, stimulating cell proliferation and collagen synthesis, thereby promoting wound healing [11]. Insufficient blood supply is considered one of the key factors leading to extensive tissue inflammation and poor soft tissue sealing around implants [12, 13]. Therefore, promoting angiogenesis in the peri-implant connective tissues is crucial to ensure effective soft tissue integration and mitigate inflammation around dental implants.

The surface morphology of materials is a pivotal physical characteristic that impacts diverse cellular behaviors, including proliferation, adhesion, and migration, through contact guidance [14, 15]. Surfaces featuring micro and nano-groove morphologies have demonstrated efficacy in modulating endothelial cell behavior and promoting vascular formation [16, 17]. However, existing material reduction technologies, such as laser etching, may adversely affect the mechanical properties of zirconia [18]. Various material additive technologies, such as the sol–gel method [19], physical vapor deposition [20] and plasma spray [21, 22], exhibit limitations such as uneven deposition, imprecise control of deposition amounts and restricted design options for coating patterns. Inkjet printing is a non-invasive technique used to deposit bioactive coatings layer by layer, enabling the formation of micron-sized patterns and structures without affecting the integrity of substrates [23]. Angiogenic growth factors and other active proteins have been applied as bioink to stimulate angiogenesis and enhance soft tissue healing [24]. However, their clinical application is limited by rapid degradation, high costs, challenging concentration control and an increased risk of infection [25]. Silk fibroin (SF) is a cost-effective and easily accessible material known for its outstanding biocompatibility and chemical stability, making it highly beneficial for biomedical applications [26]. SF can increase vascularization and reduce wound healing time in the early stages of wound repair [27]. Furthermore, SF solutions, characterized by their favorable rheological properties, serve as excellent substrates for bioprinting in various applications [28].

Given the limitations of current materials and methods for promoting angiogenesis, our study aimed to investigate the effects of silk fibroin microgroove (SFMG) structures created on zirconia surfaces using inkjet printing technology. We introduced a null hypothesis: ‘Bioactive SFMG coatings on zirconia surfaces do not enhance angiogenesis in peri-implant soft tissue compared to non-coated zirconia surfaces’. This hypothesis will be rigorously tested to evaluate the efficacy of the proposed treatments in promoting vascularization and tissue integration. Additionally, we sought to elucidate the potential intracellular signal transduction pathways of vascular endothelial cells cultured on SFMG surfaces. This study aimed to offer valuable insights into the influence and mechanisms of microgroove structures on angiogenesis, presenting a novel approach to surface design for enhancing peri-implant soft tissue integration and preventing peri-implantitis.

## Materials and methods

### Preparation of silk fibroin solution

Silkworm cocoons (5 g) were cut into small pieces and boiled in a 0.02 M sodium carbonate solution for 2 h to degum the silk. After boiling, the silk was rinsed thoroughly with ultrapure water and dried at 37°C overnight. The dried silk was weighed and immersed in 20% (w/v) lithium bromide (LiBr) solution over 72 h until completely dissolved, resulting in an amber-colored SF solution. After dialysis, the SF solution was centrifuged twice at 8000 rpm and 4°C for 20 min to eliminate impurities. The final concentration of the SF solution was established at 4% (w/v).

### Preparation of SFMG coating on zirconia

SF solution was loaded into a micro-syringe and inserted into the printing nozzle (diameter 5 µm). The SFMG coatings with widths of 30, 60 or 90 µm and depths of 5 or 10 µm (represented as 30/5, 30/10, 60/5, 60/10, 90/5 and 90/10) were fabricated on zirconia discs using the super inkjet printer (SIJ-S150, Japan) with a voltage of 600 V. The depth of the coating was regulated by the total number of printing passes: 50 cycles for achieving a 5-µm depth and 100 cycles for a 10-m depth. Smooth zirconia discs and non-groove SF-coated discs were served as the Control group and SF group, respectively. Subsequently, the printed zirconia samples were dehydrated in anhydrous ethanol for 1 h and dried overnight at 37°C.

### Characterization of SFMG coating on zirconia

The morphologies of the SFMG coatings were examined using a polarized light microscope (LB-CX, SANYO, Japan). The water contact angles (WCAs) of surfaces were measured with an optical surface tension meter (Theta, Attension, Sweden). The roughness of the zirconia coating was determined using material-laser confocal microscopy (LSM700, ZEISS, Germany). The coatings were stained with 1:1000 DAPI and examined with a Confocal Laser Scanning Microscope (CLSM, LSM780, ZEISS, Germany).

The SFMG coatings underwent ultrasonic cleaning twice for 30 min each and underwent high-pressure steam sterilization (121°C, 20 min). Fourier-transform infrared spectroscopy (FTIR) was utilized to evaluate the molecular conformation of SFMG coatings before and after this process.

### Cell culture and treatment with inhibitors

This study procured human umbilical vein endothelial cells (HUVECs) from the Cell Bank/Stem Cell Bank of the Chinese Academy of Sciences (Shanghai, China). HUVECs were cultured in an Endothelial Cell Medium (ECM, ScienCell, America) supplemented with 5% (v/v) fetal bovine serum (FBS), 1% endothelial cell growth factor (ECGF) and 1% penicillin-streptomycin (P/S). The cells were incubated in T25 culture flasks at 37°C in a humidified atmosphere with 5% CO_2_ and passaged at 80%–90% density. Cells from passages 3–6 were used for experimental purposes.

To inhibit glycolysis metabolism in HUVECs, the cells were treated with the glycolysis inhibitor 2-deoxy-d-glucose (2-DG, HY-13996, MedChemExpress (MCE)) at a concentration of 15 mM/ml for 24 h. Cells without 2-DG treatment were used as the control group.

To inhibit the activation of the PI3K-AKT pathway, the cells were treated with 2-morpholino-8-benzylchromone (LY294002, HY-10108, MCE), a PI3K inhibitor, at a concentration of 10 µM/ml for 24 h. Cells without LY294002 treatment were used as the control group.

### Adhesion of HUVECs cultured on SFMG coatings

HUVECs were seeded onto zirconia disks placed in 24-well cell culture plates at a density of 5 × 10^4^/well and incubated for 3 days. Following incubation, the cells were rinsed thrice with PBS and then fixed in 2.5% glutaraldehyde for 2 h at room temperature. Subsequently, dehydration was conducted sequentially using ethanol with concentrations of 50%, 70%, 80%, 90% and two rounds of 100%. Scanning electron microscopy (SEM) was employed to observe cellular morphology.

### The cell cytoskeleton of HUVECs cultured on SFMG coatings

HUVECs were cultured on zirconia disks in 24-well cell culture plates. The cells were rinsed thrice with PBS and then fixed with 4% paraformaldehyde (PFA) for 20 min. Subsequently, cells were permeabilized with a 0.2% Triton™ X-100/PBS solution for 5 min. For cytoskeletal visualization, cells were stained with fluorescein isothiocyanate (FITC)-labelled phalloidin (Solarbio, China, 1:50), and nuclei were counterstained using DAPI (Beyotime, China, 1:100). Representative fluorescence images were captured utilizing a CLSM (LSM780, ZEISS, Germany).

### Cell proliferation assay

Cell proliferation of HUVECs cultured on different zirconia disks was quantitatively assessed using the CCK-8 assay. Cells were seeded on zirconia disks within 96-well cell culture plates at a 5 × 10^3^/well density. At 1, 3 and 7 days after seeding, cells were rinsed three times with PBS. Subsequently, 100 μl fresh ECM medium (no FBS) containing 10 μl of CCK-8 reagent (CK04, Dojindo, Japan) was added to each well. Cells were incubated at 37°C for another 2 h. Finally, 100 μl medium was collected to measure the absorbance at 450 nm.

Additionally, another set of cells was seeded in 96-well plates at a 5 × 10^3^/well density and cultured for 24 h. The EdU assay kit (KeyGEN biotech, China) was used following the manufacturer’s instructions. After introducing EdU to the cultured cells and subsequent incubation, cells were fixed and labeled, and a fluorescent dye was added. Subsequently, nuclei were counterstained with DAPI, and fluorescent images were captured using CLSM (LSM780, ZEISS, Germany). Proliferation rates were quantified by calculating the ratio of EdU-positive to total cells.

### Implanting surgery and histological examination

Animal experiments were conducted with approval from the Institutional Animal Care and Use Committee (IACUC) at Sun Yat-sen University (approval no. SYSU-IACUC-2022-001406), adhering to the guidelines set by the Animal Ethical and Welfare Committee of Sun Yat-sen University. Twenty-four male Wistar rats (8 weeks old, mean body weight 250 g) were anesthetized using 5% pentobarbital sodium (0.3 ml per 100 g intraperitoneal injection) before treatment. Initially, the bilateral maxillary first molars were extracted. Implant sockets were prepared with continuous saline irrigation using a P drill (20-I20), followed by the insertion of mini-implants with zirconia abutments. Pain management and infection prevention were addressed by administering buprenorphine (0.05 mg/kg via intraperitoneal injection) and prophylactic clindamycin. The rats were provided with powdered food until the time of euthanasia. After healing for 4 weeks, rats were sacrificed with an overdose of phenobarbitone, and both the mini-implants and the surrounding soft tissues were excised. The excised samples were initially fixed in 4% paraformaldehyde (PFA, Beyotime, China) and then subjected to decalcification in a solution (containing 10% EDTA and 4% sucrose in 0.01 M PBS) at 4°C for 5 days to remove the buccal gingival tissue and separate the implant from the soft tissue of the jawbone. The soft tissue on the palatal side was removed, rinsed, dehydrated, embedded and sectioned into 5 µm thick slices using a Leica Biosystems RM2255 (Leica, Germany). The obtained sections were then subjected to H&E and Masson staining, and the stained images were captured using a digital slide scanning system (Olympus, Japan).

### Immunofluorescence staining

Immunohistochemistry staining was conducted using the UltraSensitive SP immunohistochemistry kit (Fuzhou Maixin Biotechnology, Ltd, China) according to the manufacturer’s guidelines. Briefly, the samples underwent treatment with 3% H_2_O_2_ followed by antigen retrieval with 0.1% trypsin and blocking with 5% (w/v) bovine serum albumin (BSA) (Beyotime, China). The sections were then incubated with primary antibodies against CD31 (1:100 in PBS, Beyotime, China) overnight at 4°C. After rinsing with PBS, the sections were incubated with appropriate biotinylated secondary antibodies and horseradish peroxidase-conjugated streptavidin-biotin. The presence of endothelial cells was estimated by calculating the number of CD31-positive cells. Three random views were examined using ImageJ 1.8.0 software to determine the mean optical density (MOD) value of CD31. Gray values were converted to standard optical density values for the analysis, enabling the calculation of positively stained areas.

### Quantitative real-time PCR analysis

Cells were seeded on zirconia disks in 6-well cell culture plates with a density of 2 × 10^5^ and cultured for 72 h. Total RNA was extracted utilizing the MolPure^®^ Cell/Tissue miRNA Kit (Yeasen Biotechnology, China), following a protocol that included cell lysis, RNA binding, washing and elution steps provided by the manufacturer. The isolated RNA was then reverse-transcribed into complementary DNA (cDNA) using the Takara Prime Script TM RT reagent Kit (#RR047A; Agilent Technologies, United States) according to the manufacturer’s recommendation. Subsequently, real-time polymerase chain reaction (PCR) was conducted using Hieff^®^ qPCR SYBR Green Master Mix (No Rox) (Yeasen Biotechnology, China) with denaturation, annealing and extension cycles according to the manufacturer’s protocol. The primer sequences for the target genes are listed in Table 1.

**Table 1.** The primer sequences for the target genes

Genes	Primers	Sequences
VEGF-A	Forward Reverse	AGGGCAGAATCATCACGAAG GGATGGCTTGAAGATGTACTCG
bFGF	Forward Reverse	GTGGATTCTGCTTCCCCTG TGATTTCCCCTTCAGCCATG
PI3K	Forward Reverse	GGAGCTGGCTACTTCTCGC GGGAACATCCTCCTTCAACAG
HK2	Forward Reverse	GAGCCACCACTCACCCTACT CCAGGCATTCGGCAATGTG
PKM2	Forward Reverse	ATGTCGAAGCCCCATAGTGAA TGGGTGGTGAATCAATGTCCA
PGK1	Forward Reverse	TGGACGTTAAAGGGAAGCGG GCTCATAAGGACTACCGACTTGG
LDHA	Forward Reverse	ATGGCAACTCTAAAGGATCAGC CCAACCCCAACAACTGTAATCT
PFKFB3	Forward Reverse	TTGGCGTCCCCACAAAAGT AGTTGTAGGAGCTGTACTGCTT
GAPDH	Forward Reverse	TTGCAGTGGCAAAGTGGAGA GATGGGCTTCCCGTTGATGA

### Protein extraction and western blotting

Cells lysis was performed using RIPA lysis buffer containing 1% protease-phosphatase inhibitors followed by centrifugation at 15 000 g for 20 min at 4°C. Protein concentrations were determined using a BCA protein assay kit (Cwbio, China). Subsequently, cell lysates were mixed with sodium dodecyl sulfate (SDS) sample loading buffer (#20315ES05; Yeasen Biotechnology, China) and boiled for 5 min at 95°C. Then, 50 ng of protein was separated by SDS-PAGE and transferred to PVDF membranes. The PVDF membranes were blocked with 5% BSA for 1 h at room temperature. After rinsed three times with PBS, the PVDF membranes were incubated in the primary antibodies of β-tubulin (1:1000, EMRR, China, EM32016-01), GAPDH (1:5000, Proteintech, China, 60004-1-Ig), vascular endothelial growth factor (VEGF-A) (1:1000, Affinity, USA, #AF5131), fibroblast growth factor (bFGF) (1:1000, Affinity, USA, #DF8038), PI3K (1:1000, Cell Signaling Technology (CST), America4257T), p-AKT (1:1000, CST, America, 4060 T), p-mTOR (66888-1-Ig, Proteintech, China) at 4°C overnight. After three additional PBS washes, the PVDF membranes were exposed to respective secondary antibodies (Jackson, USA, 1:8000) at room temperature for 1 h. Finally, the protein blots were visualized using an electrochemiluminescence reagent (ChemiDoc, Bio-Rad, USA).

### Collection of HUVECs culturing supernatants

HUVECs were seeded on different zirconia disks in 24-well cell culture plates at a density of 5 × 10^4^/well. The cells were cultured in ECM devoid of FBS and ECGF. After 12 h of incubation, supernatants from each experimental group were collected and centrifuged at 1000 rpm for 15 min. The resulting supernatants were promptly utilized or stored at −80°C for future use.

### ELISA assay

The concentrations of VEGF-A and bFGF proteins in cell culture supernatants were quantified using commercial ELISA kits (Cusabio, China). Samples were applied to microplates pre-coated with specific antibodies, followed by incubation with horseradish peroxidase (HRP)-conjugated detection antibodies. After the colorimetric reaction, the absorbance was measured at 450 nm using a microplate reader. Protein concentrations were calculated based on a standard curve.

### Transwell assay

The Transwell assay was utilized to assess the impact of SFMG coatings on HUVEC migration. Briefly, 200 µl of cell suspension with a density of 5 × 10^4^ cells/well, prepared using medium without FBS, was added to the upper chambers of a 24-well Transwell plate (Corning; pore size = 8 μm). Subsequently, 600 µl of supernatants from different experimental groups were added to the lower chambers. The cells on the upper filter surfaces of the upper chambers were removed with a cotton swab. The lower surface of the filter was fixed with 4% PFA and stained with 0.1% crystal violet for 10 min. The microscope evaluated five randomly selected fields per filter (OLYMPUS, Japan).

### Tube formation assay

Matrigel™ matrix (BD Bioscience, USA) was coated onto the bottom of each well in a 48-well plate using 100 µl per well. Cells were seeded into each well at a density of 5 × 10^4^/well and then treated with different supernatants from each group. After 8-h incubation, the nodules were counted with a microscope. The length of circular and tubular structures formed within the Matrigel was quantified for analysis.

### RNA sequencing and data analysis

HUVECs were cultured on zirconia discs in 6-well cell culture plates at a density of 2 × 10^5^ cells per well for 72 h. The cells were lysed using Trizol (Beyotime, China), and the resulting lysates were submitted to Nanjing Persomics Genetech Company Limited for mRNA isolation, Illumina sequencing library construction, double-stranded cDNA synthesis, bioinformatics analysis and high-throughput sequencing. Each group included three biological replicate samples. Differentially expressed genes (DEGs) were identified based on a log 2-fold change (FC) > |1| and *P* values < 0.01. To elucidate their biological significance, the expression of related differential genes was analyzed using a heatmap, Gene Ontology (GO) analysis and Kyoto Encyclopedia of Genes and Genomes (KEGG) pathway analysis (threshold: *P* values < 0.05).

### Statistical analysis

Data were expressed as mean ± SD by using GraphPad Prism 9.0 software. Multiple group comparisons were performed by one-way ANOVA. Statistical significance was determined at *P *<* *0.05.

## Results

### Characterization of SFMG coating on zirconia

**Figure 1.**
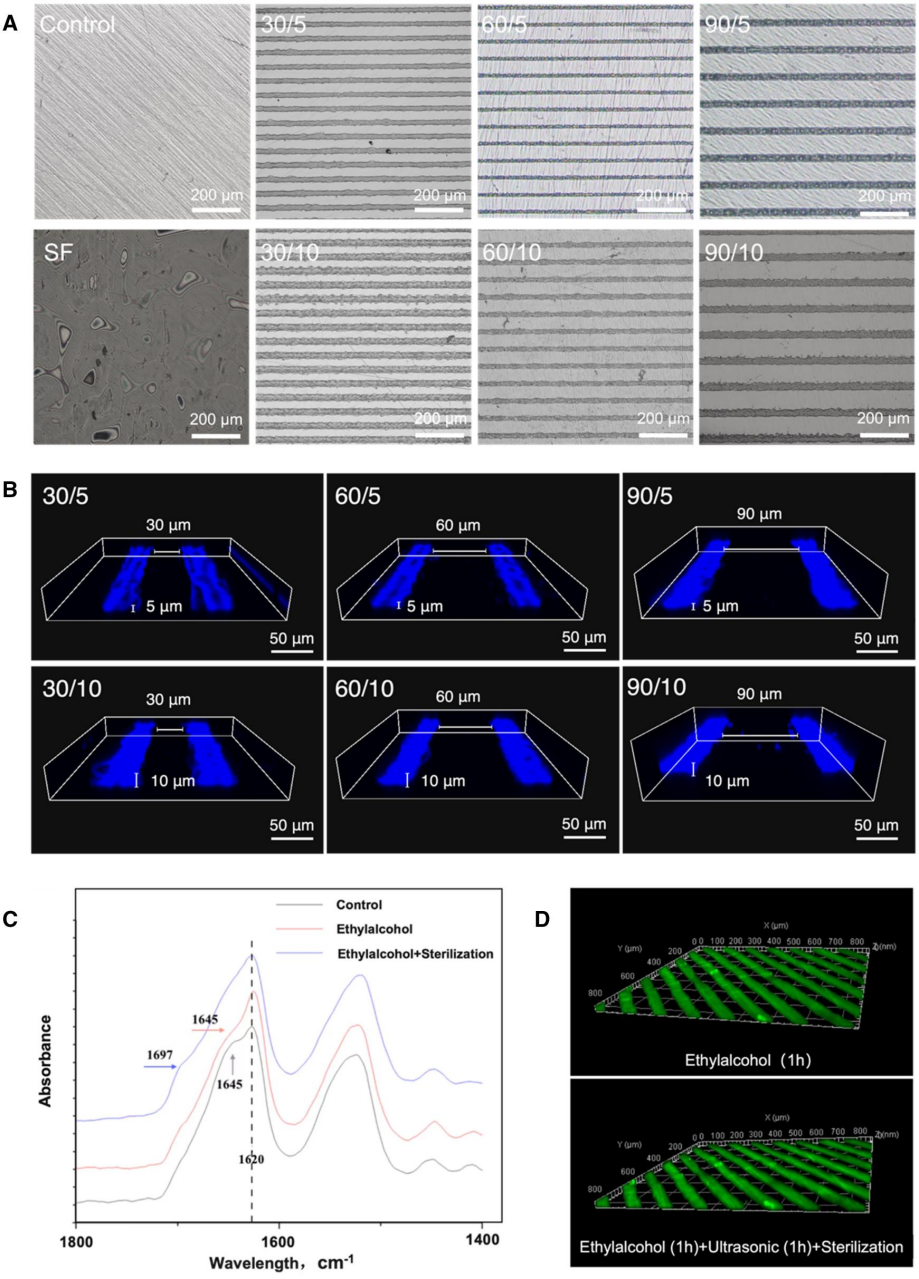
Morphology of SF microgrooves coating on zirconia. (**A**)The morphology SFMG coatings with different sizes detected by polarized light microscopy (bar scale = 200 μm). (**B**) The widths and depths of SFMG showed in the 3D reconstruction images of SFMG coatings (CLSM, bar scale = 50 μm). (**C**) FTIR spectra of SFMG coatings of control group (without dehydrated), ethyl alcohol group (dehydrated in absolute ethanol for 1 h) and ethyl alcohol + sterilization group (high-pressure steam sterilization after dehydrated in absolute ethanol for 1 h). Dotted line indicates the main peak, and arrows denote shoulder peaks. (**D**) The 3D SFMG images before (top) and after (bottom) ultrasonication and sterilization treatment.

CLSM were utilized to evaluate the surface morphology of zirconia surfaces coated with SFMGs. All examined groups demonstrated uniform and consistent SFMG coatings of similar dimensions (Figure 1A). The CLSM images presented 3D reconstructions of SFMGs with widths of 30, 60 or 90 µm, and depths of 5 or 10 µm (Figure 1B).

Prior to clinical application, abutments often require autoclaving for sterilization. Additionally, ultrasonic agitation is commonly employed during routine cleaning procedures to remove debris and impurities from the surface of the abutments before they are subjected to high-pressure steam sterilization. Therefore, we assessed the physical and chemical stability of SFMG structures to consider clinical application. The FTIR results showed that both untreated and treated SF coatings displayed a distinct peak at 1620 cm^−1^, indicating the presence of β-sheet folding. However, the untreated SFMG coating exhibited an additional shoulder peak at 1645 cm^−1^, suggesting an irregular coil structure. Post-treatment with anhydrous ethanol, the SFMG coating exhibited enhanced absorption at 1620 cm^−1^ and a reduced shoulder peak at 1645 cm^−1^. Subsequent high-pressure steam sterilization further strengthened the peak at 1620 cm^−1^ and introduced a distinctive peak at 1697 cm^−1^, representing β-sheet folding (Figure 1C). CLSM 3D reconstructions revealed no significant alterations in the morphology, including width and depth, of SFMG coatings following ultrasonic agitation and high-pressure steam sterilization (Figure 1D).

**Figure 2.**
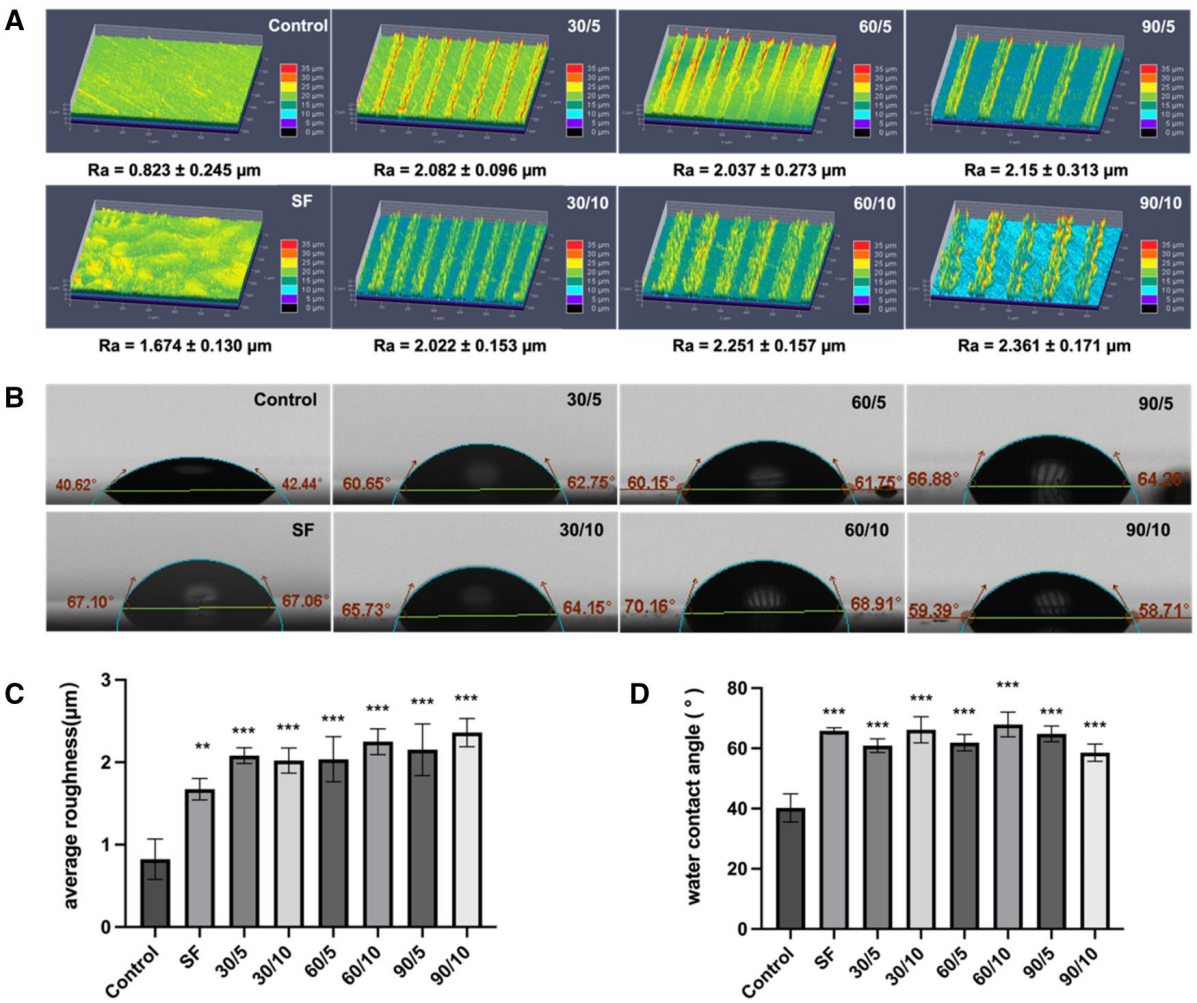
The hydrophilic and surface roughness of SFMG. Representative images of roughness (**A**) and WCAs (**B**) of SFMG coatings on zirconia surfaces. Quantitative results of roughness (**C**) and WCAs (**D**) of SFMG coatings on zirconia surfaces (***P < 0.001, **P < 0.01, *P < 0.05).

Comparatively, all surfaces of both SF and SFMG groups exhibited significantly greater roughness than the smooth zirconia surface, with the highest roughness observed in the 90/10 group (Figure 2A and C). Additionally, the WCA of the zirconia surface in the control group was approximately 40°, while the WCA of SF-coated groups ranged from 60° to 70° (Figure 2B and D).

### Morphology of HUVECs on the SFMG coating on zirconia

**Figure 3.**
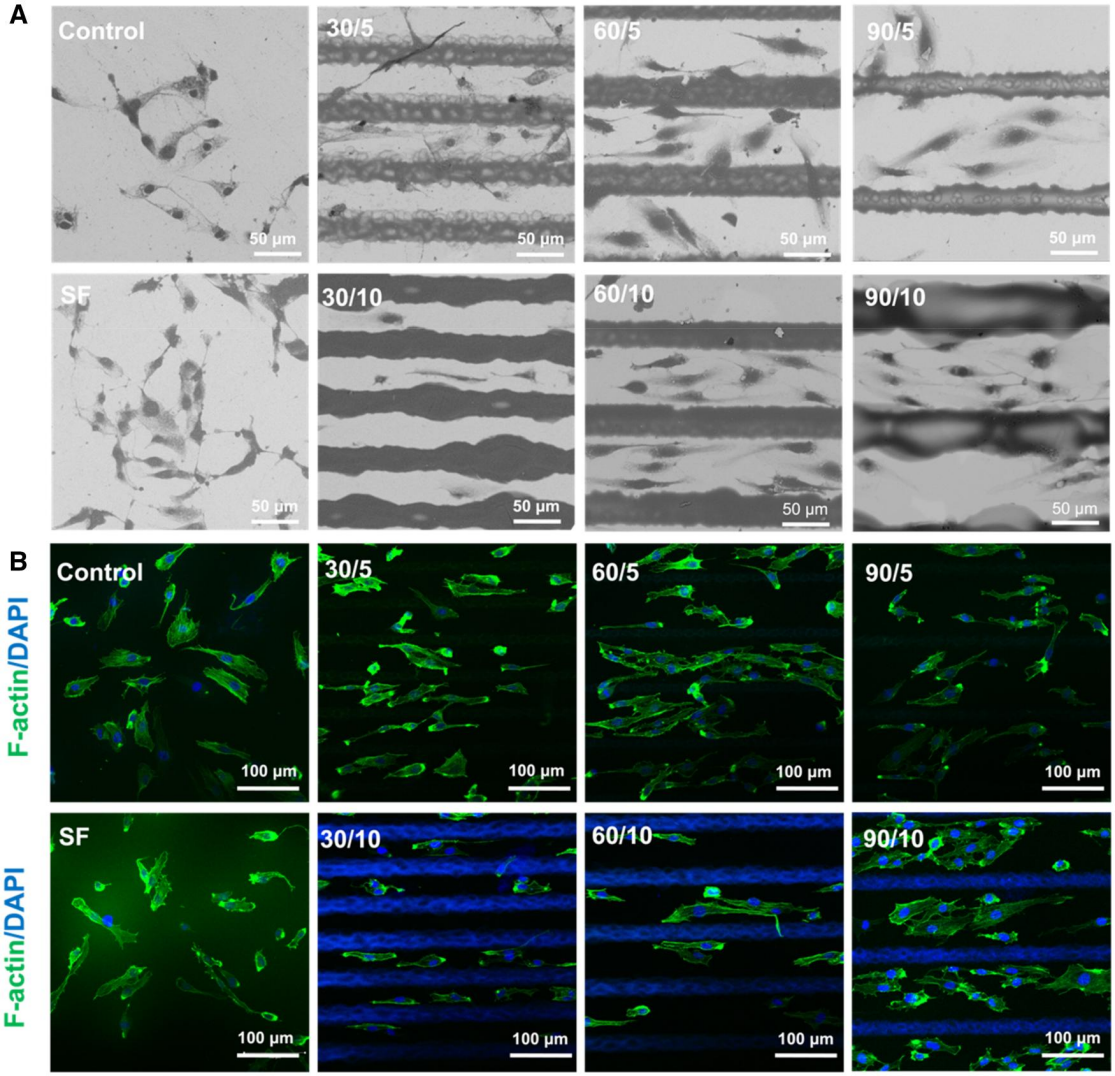
The morphology of HUVECs cultured on the SFMG-coated surfaces. (**A**) SEM images of HUVECs after culturing for 3 days on zirconia surfaces (bar scale = 100 µm). (**B**) Immuno-fluorescent images of HUVECs cultured on zirconia discs for 24 h (CLSM, bar scale = 100 µm).

SEM (Figure 3A) and CLSM (Figure 3B) were employed to visualize the cellular morphology of endothelial cells on different groups. HUVECs cultured in the SF group and the control group exhibited a random and non-directional arrangement, whereas cells cultured on the SFMG-coated surfaces presented a structured alignment along the long axis of the grooves. Additionally, cells in the SFMG-coated group displayed an elongated, spindle-shaped morphology. Notably, in the 5 µm deep groove-coated group, some cells adhered to the top of groove surfaces. In the 10 µm deep groove-coated groups, cells exhibited spindle-shaped morphology attached at the bottom of grooves.

### SFMG zirconia surfaces promoted the proliferation of HUVECs

**Figure 4.**
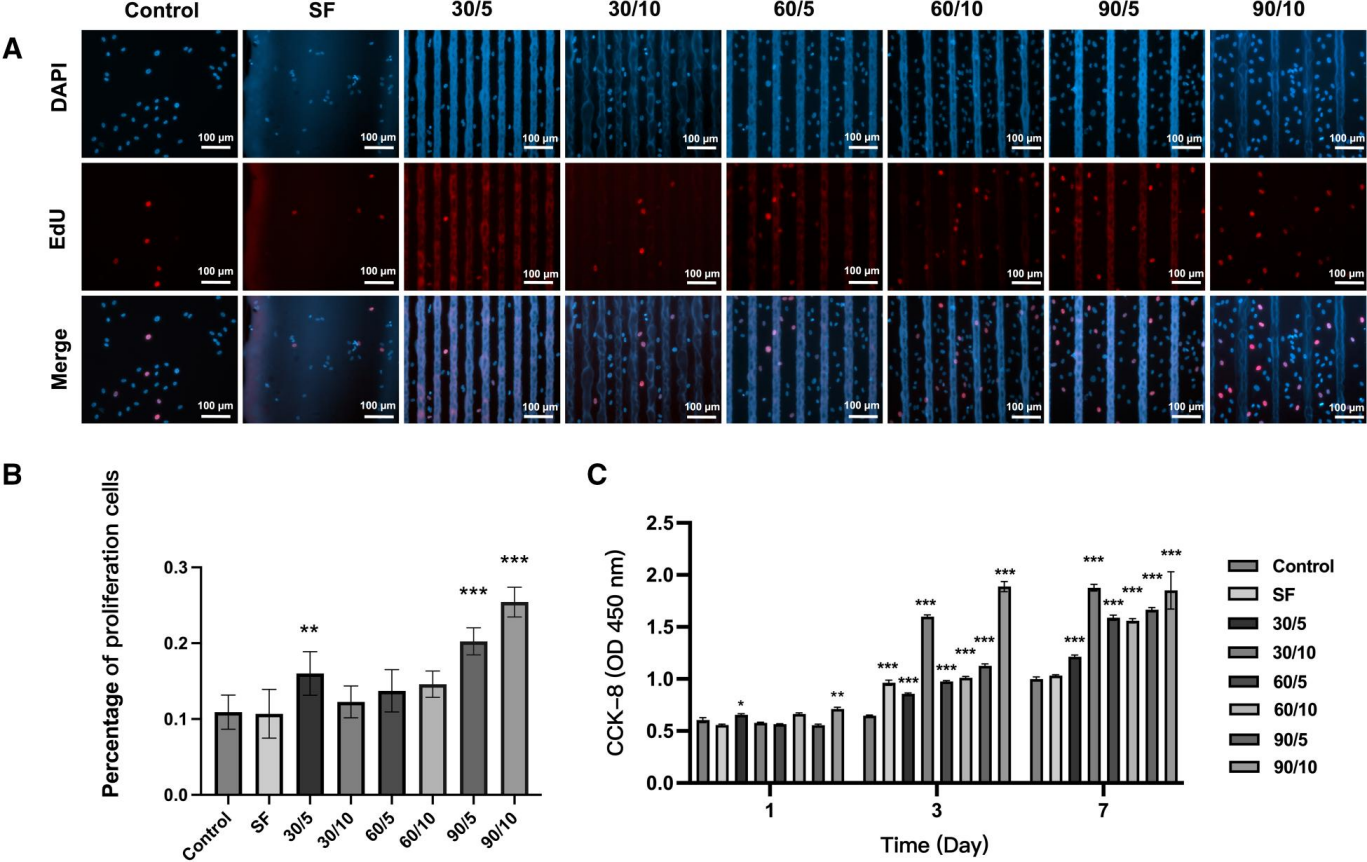
SFMG zirconia surfaces promoted the proliferation of HUVECs. (**A**) EdU staining images of HUVECs cultured on different groups for 24 h (bar scale = 100 µm). (**B**) Semiquantitative analysis of the EdU staining assay. (**C**) Absorbance measurements for CCK-8 results of HUVECs proliferation after culturing on different groups for 1, 3 and 7 days (***P <* *0.001, **P <* *0.01, *P <* *0.05).

After a 24-h incubation period, the cells in the 90/10 SFMG group displayed the most significant increase in proliferation (Figure 4A and B). The CCK-8 results showed that all SFMG coatings facilitated a more effective proliferation of HUVECs compared to the control group, highlighting the remarkable biocompatibility of SFMG coatings. Among them, the 30/10 and 90/10 groups exhibited the most notable cell proliferation-promoting effects (Figure 4C).

Notably, the 90/10 SFMG coating group exhibited superior endothelial cell growth and proliferation enhancement. Consequently, the 90/10 group was identified as the optimal SFMG coating composition for further exploration of the biological impacts of SFMG morphology on endothelial cells.

### SFMG zirconia surfaces promoted the migration and angiogenesis of HUVECs

**Figure 5.**
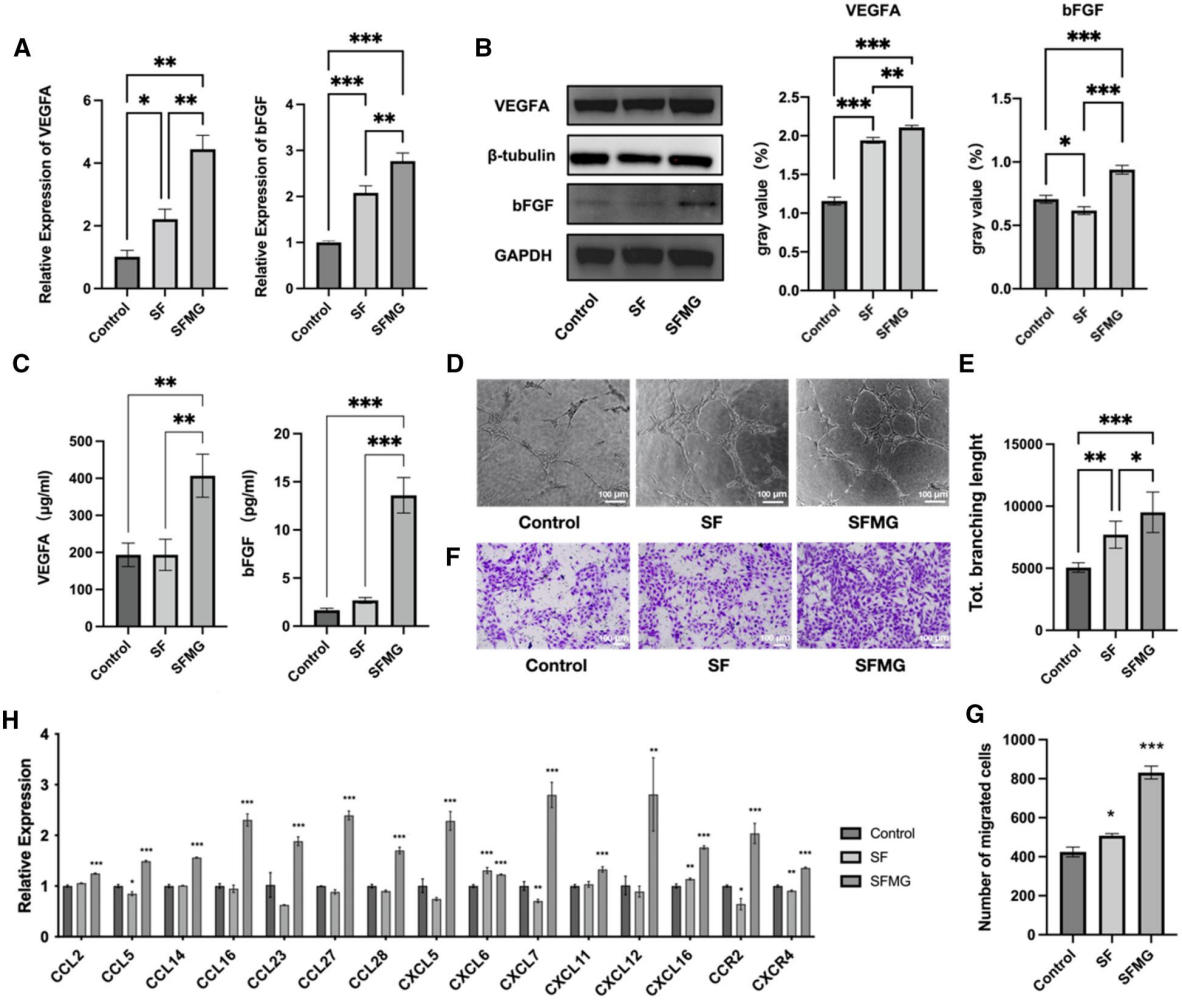
SFMG zirconia surfaces promoted the migration and angiogenesis of HUVECs. (**A**) The q-PCR results of the expression of VEGF-A、bFGF. (**B**) Representative bands and grayscale analysis of VEGF-A, bFGF western blot. (**C**) ELISA results of secreted VEGF-A, bFGF protein. (**D**) Representative images of tube formation (bar scale = 100 µm). (**E**) Semi-quantitative statistical analysis of the total branching length. (**F**) Representative images of Transwell assay results (crystal violet stain, bar scale = 100 µm). (**G**) Semi-quantitative statistical analysis of the number of migrated cells. (**H**) The q-PCR results of the expression of chemokines (***P <* *0.001, **P < 0.01, *P <* *0.05).

HUVECs cultured on SFMG-coated surfaces exhibited significantly elevated expression of VEGF-A and bFGF genes and proteins compared to control groups, resulting in heightened VEGF-A and bFGF synthesis within HUVECs (Figure 5A and B). ELISA assays on cell culture supernatants collected after 24 h revealed notable elevations in the secretion of VEGF-A and bFGF by HUVECs grown on the SFMG-coated surfaces (Figure 5C). Moreover, tube formation assays exhibited more extensive luminal structures with longer and wider tubes in the SFMG group (Figure 5D and E). Transwell experiments were performed to assess the directional migration ability of HUVECs influenced by culture supernatants from different groups. Notably, the supernatants from the SFMG group significantly enhanced HUVEC migration through the pores towards the lower chamber, resulting in increased cell numbers (Figure 5F and G). Chemokines, pivotal for cell migration, are essential in the initial stages of angiogenesis, guiding endothelial cells to vascular formation sites [29, 30]. The SFMG group showed an upregulation in the expression of various cell chemokines in HUVECs (Figure 5H).

### SFMG zirconia surfaces promoted angiogenesis of the peri-implant abutment connective tissue

**Figure 6.**
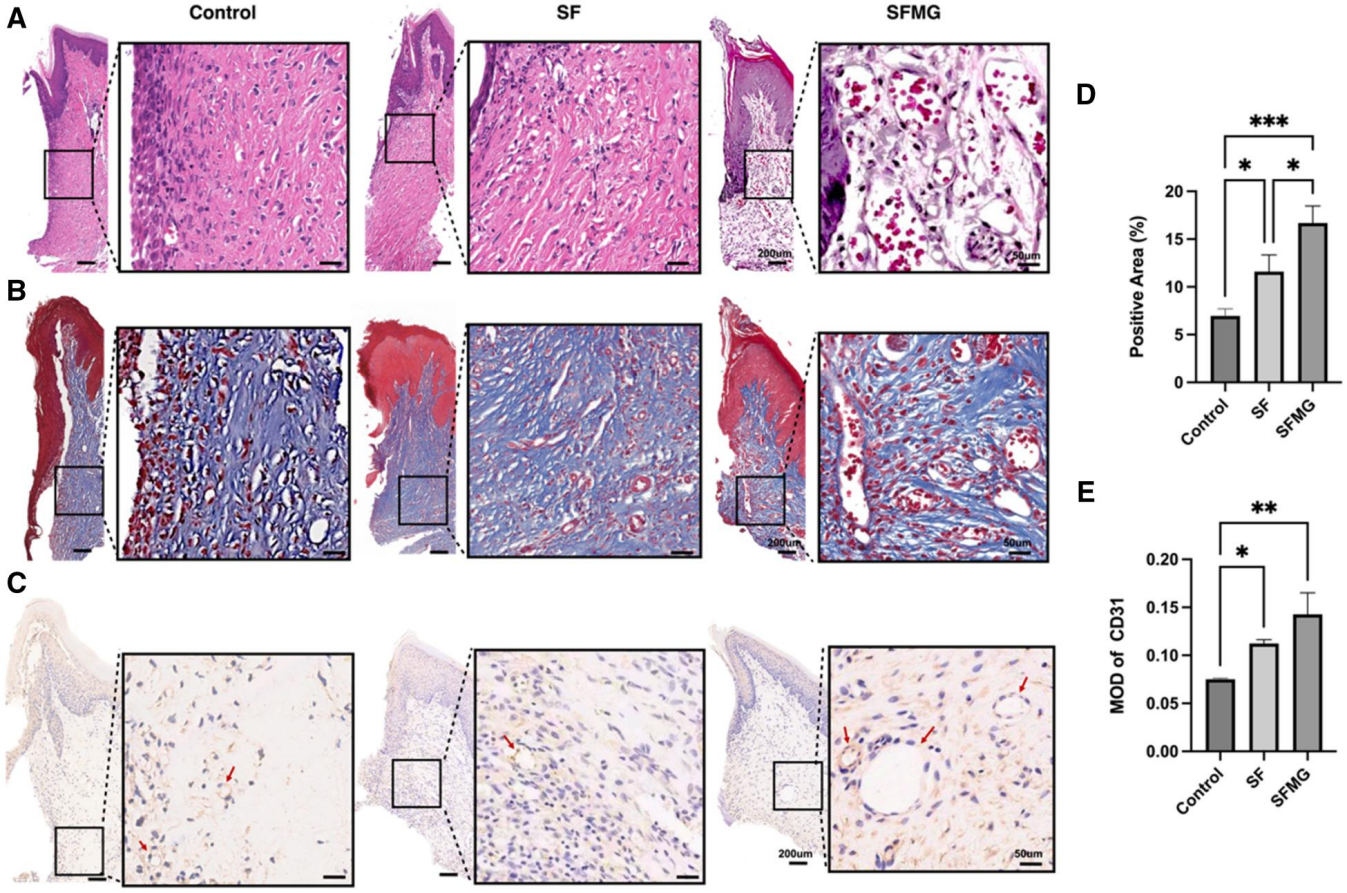
SFMG zirconia surfaces promoted angiogenesis of the peri-implant abutment connective tissue. Representative images of (**A**) H&E staining, (**B**) Masson staining and (**C**) IHC staining of CD31. Bar scale = 200 μm. Quantitative analysis of the fraction of positively stained areas (**D**) and mean option density (MOD) (**E**).

As shown in the H&E-stained and Masson-stained histology sections, there was no significant difference in the vascular density between the control group and SF group. Nevertheless, notably elevated blood vessel density, accompanied by larger luminal structures, was observed in the connective tissues surrounding the SFMG compared to the control group (Figure 6A and B). Furthermore, more blood vessels and CD31-positive stained cells were detected in the SFMG group compared to the control and SF groups (Figure 6C–E).

### The effects of SFMG on HUVECs were glycolytic dependent

**Figure 7.**
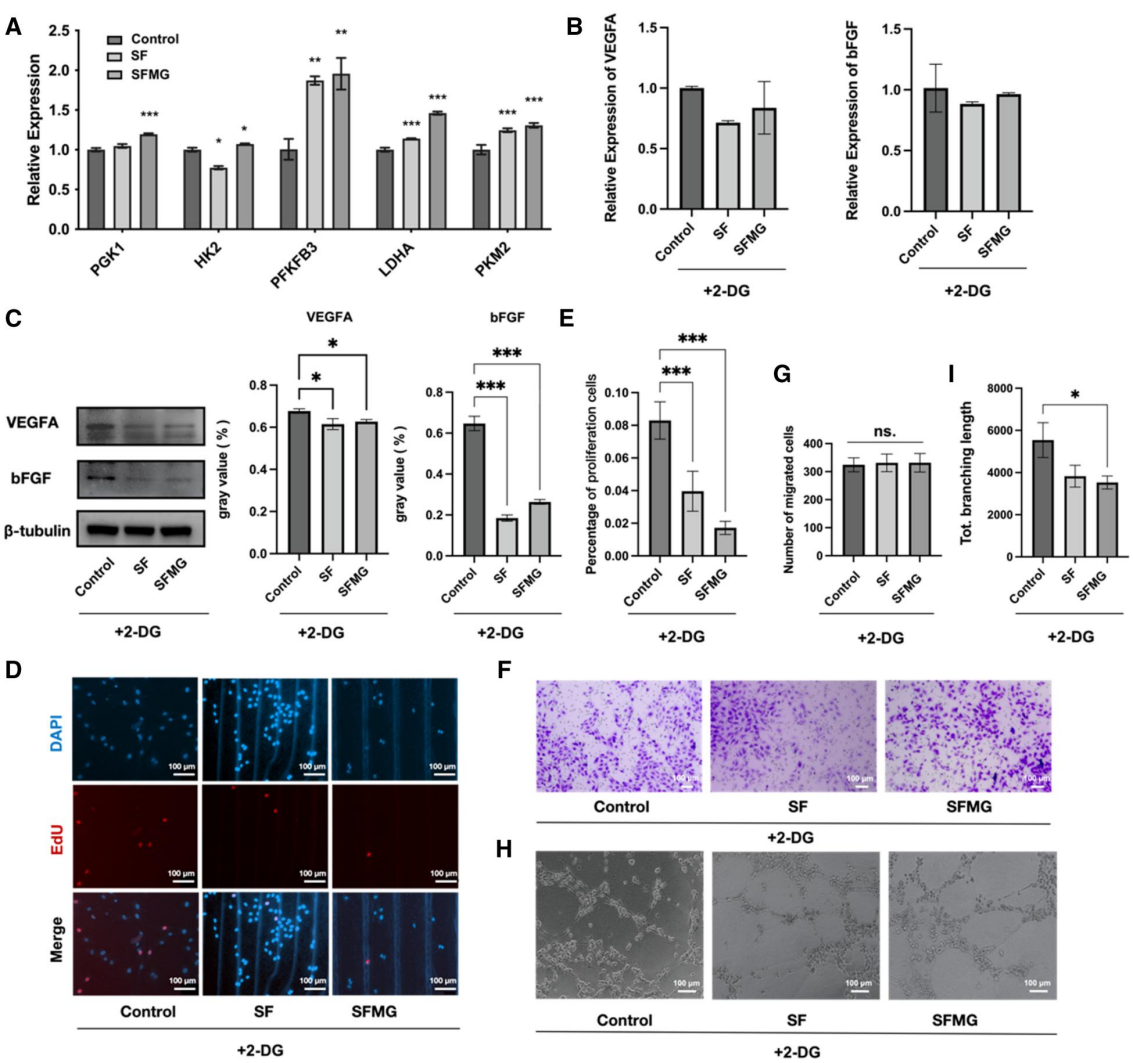
The effects of SFMG on HUVECs were glycolytic dependent. (**A**) The q-PCR results of the expression of key enzymes involved in glycolysis. (**B**) The q-PCR results and (**C**) western blot results of the expression of VEGF-A, bFGF after 2-DG treatment. (**D**) Representative images of EdU staining (CLSM) and (**E**) cell proliferation rate after 2-DG treatment. (**F**) Representative images of Transwell assay results and (**G**) the number of migrated cells after 2-DG treatment. (**H**) Representative images of tube formation and (**I**) total branching length after the addition of 2-DG (***P <* *0.001, **P <* *0.01, *P <* *0.05).

qRT-PCR was employed to assess the gene expression of key glycolytic enzymes in HUVECs. The gene expression of glycolytic enzymes (HK2, PGK1, PFKFB3, PKM2, LDHA) was significantly upregulated in HUVECs of the SFMG group (Figure 7A). However, upon the introduction of 2-DG, an inhibitor of glycolysis, the previously observed enhancement in the expression of VEGF-A and bFGF, as well as the proliferation, migration and tube formation capacities of HUVECs stimulated by SFMG coatings, was diminished (Figure 7B–H).

### SFMG zirconia surfaces regulated HUVECs glycolysis through PI3K-AKT pathway

**Figure 8.**
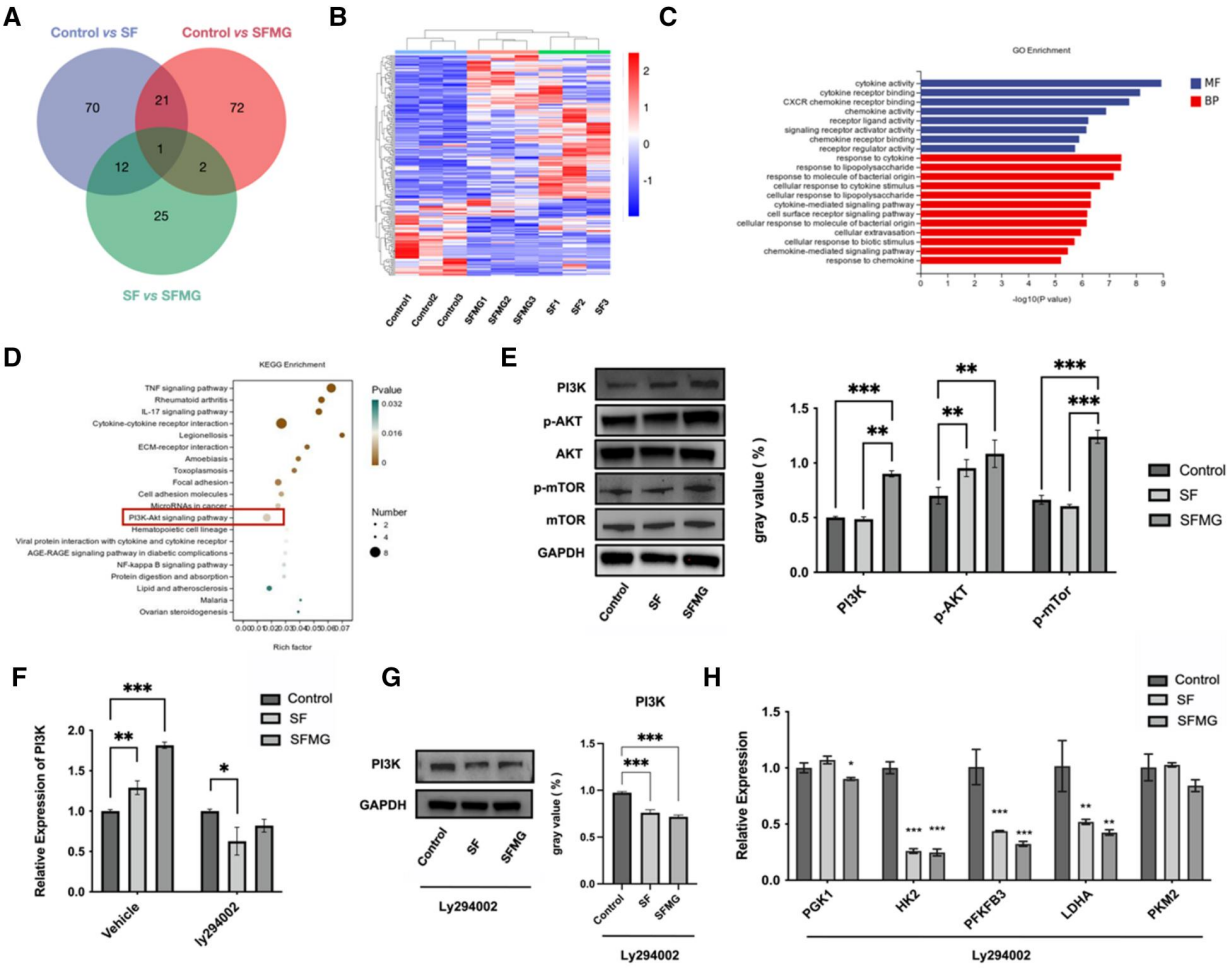
SFMG zirconia surfaces regulated HUVECs glycolysis through PI3K-AKT pathway. (**A**) Venn diagram of DEGs among the groups. (**B**) Heatmap of co-expressed DEGs among the groups. (**C**) Up-enrichment GO bar plots of SFMG group versus control group (MF: molecular function, BP: biological process). (**D**) KEGG pathway enrichment analysis of SFMG group versus control group. (*X*-axis, gene ratio; *Y*-axis, enriched pathways; dot sizes, the number of differential expressed cytokines in the enriched pathway; dot color, adjusted *P* values.) (**E**) Western blot results of the proteins related in PI3K-AKT signaling pathway. (**F**) The gene expression of PI3K in HUVECs with and without PI3K inhibitor treatment. (**G**) The expression of PI3K protein in HUVECs after PI3K inhibitor treatment. (**H**) Expression of key glycolytic enzyme genes in HUVECs after PI3K inhibitor treatment (***P < 0.001, **P < 0.01, *P < 0.05).

The Venn diagram illustrated the overlapping DEGs among the groups (control vs. SF, SF vs. SFMG, and SFMG vs. control) (Figure 8A). Hierarchical clustering heatmap identified DEGs among the groups (Figure 8B). GO analysis revealed enrichment related to cytokine activity, cytokine receptor binding, CXCR chemokine receptor binding, response to cytokine, and response to chemokine (Figure 8C). These findings indicated that it significantly influences intercellular signaling processes. Moreover, the KEGG analysis indicated an upregulation of DEGs associated with the PI3K/AKT signaling pathway in the SFMG group (Figure 8D).

The SFMG group showed increased expression levels of the PI3K gene and protein phosphorylation of AKT and mTOR (Figure 8E). Additionally, after inhibiting PI3K activity by adding a PI3K pathway inhibitor (Ly294002), the expression of glycolytic enzymes (PGK1, HK2, PFKFB3, LDHA, PKM2) in HUVECs was significantly suppressed (Figure 8F–H).

## Discussion

As posited, the null hypothesis suggested no significant difference in angiogenesis between the treated and untreated zirconia surfaces. Contrary to this hypothesis, our findings demonstrated a marked increase in VEGF expression and fibroblast growth factor in HUVECs cultured on bioactive SFMG-coated surfaces. These results not only refute the null hypothesis but also highlight the potential of SFMG structure in enhancing peri-implant soft tissue integration and vascularization. This refutation underscores the effectiveness of the SFMG coatings in facilitating significant biological improvements over the standard zirconia surfaces.

Zirconia abutments are extensively utilized in clinic settings due to their superior aesthetic properties and reduced bacterial adhesion. However, the biologically inert characteristics of zirconia surfaces can lead to insufficient integration with the surrounding soft tissues [31]. Proper vascular remodeling and neoangiogenesis are essential for promoting healing and preventing scar tissue formation around dental implants [1]. Therefore, improving the surface properties of zirconia abutments to facilitate peri-implant vascularization is a critical issue requiring immediate attention.

Currently, angiogenesis promotion methods mainly rely on chemical modification of materials. However, chemical approaches, such as incorporating growth factors, face challenges such as high costs, ethical debates and complex synthesis processes, resulting in local immune reactions at the wound site [32]. In contrast, physical modification methods offer a promising alternative, providing convenient synthesis, ensuring safety and stability and avoiding the issues associated with chemical methods. Crucially, the surfaces morphology of materials plays a crucial role as a physical feature influencing various cell behaviors through contact guidance, where surface patterns direct cell movement, enhancing bioactivity, growth and differentiation [33]. Various reported material surface morphologies include regular patterns like linear grooves, microcolumns, micropores, nanotubes and irregular patterns characterized by surface roughness. A systematic review demonstrated that optimal proliferation of gingival fibroblasts occurred on zirconia and titanium surfaces featuring nanotubules or microgrooves [34]. Linear microgroove patterns have been shown to promote the proliferation and angiogenesis of endothelial cells by facilitating the alignment and growth along the groove direction [35]. However, the brittleness of zirconia poses challenges, where mechanical processing for creating microgrooves on titanium surfaces can lead to the cracking of zirconia abutments, increasing the risk of failure [36]. Inkjet printing technology, recognized for its simplicity and non-invasive deposition capabilities, emerges as a promising strategy for surface modification. Common bio-inks such as collagen [37], alginate [38], gelatin methacryloyl (GelMA) [39] and photopolymerizable resins [40] have been extensively used due to their utility in various applications. However, these materials often encounter challenges like rapid degradation, inadequate mechanical properties or insufficient bioactivity, which can limit their practical applications. Among various bio-inks, SF stands out due to its wide-ranging applications, attributed to its structural versatility and amenability to various processing techniques. This allows for the fabrication of complex, functionally graded structures [41]. A recent study highlighted the capability of SF to form patterned films, which guide the behavior of HUVECs and demonstrate its potential in vascular tissue engineering [42]. Furthermore, the precise microstructural control achievable with SF enhances its application in neural tissue engineering, where aligned fibroin structures promote neuronal growth and connectivity [43]. Such alignment is crucial for the functional regeneration of complex tissues, underscoring the transformative potential of SF in bioprinting applications. In this experiment, to maintain the mechanical integrity of zirconia, inkjet printing using SF as the bio-ink was employed to prepare microgroove bio-coatings layer by layer on the surface of zirconia, thereby enhancing the biological performance of zirconia abutment surfaces. By adjusting the parameters of inkjet printing and controlling the number of printed layers and spacing, precise control over the groove dimensions was achieved. The SEM and CLSM images revealed the precisely controlled dimensions of the microgrooves in terms of depth and width. This level of control can be attributed to the moderate fluidity and excellent printability of the SF solution [44].

Given the operational demands on dental implant abutments, bioactive coatings must resist routine cleaning and sterilization. This study demonstrated that the zirconia SFMG coating retained its appearance after undergoing ultrasonic agitation and high-pressure steam sterilization, with a molecular shift from irregular random coils to β-folding. *In vitro* studies have shown that β-folding structures exhibit greater molecular density [45], and a higher concentration of these structures enhances the stability of SF, resulting in a lower degradation rate [46]. Thus, these findings collectively suggest that the SFMG coating exhibits excellent physical adhesion to the zirconia surface, withstands mechanical stress and shows remarkable thermal stability within its structural characteristics.

Various studies have demonstrated the efficacy of rough surface morphology in regulating the proliferation of vascular cells [47]. Moreover, Van Wachem *et al.* [48] highlighted the positive impact of moderately hydrophilic polymers on promoting endothelial cell growth. It is consistently shown in research that HUVECs display optimized adhesive properties when the WCA ranges from 60° to 70° [49]. In this study, our results indicated that the SFMGs coatings prepared on zirconia surfaces significantly increase surface roughness while maintaining moderate hydrophilicity, potentially enhancing cell adhesion and proliferation.

The effects of microgroove dimensions on cell specificity have yielded inconsistent conclusions due to factors such as material diversity, fabrication methods, morphological sizes and cell type choices. Studies have shown that groove widths ranging from 750 to 100 μm can effectively enhance the functionality of endothelial cells on titanium surfaces [50]. Another study observed that microgroove structures, formed by nanofiber scaffold materials with widths of 15, 50 and 100 μm, and depths of 2, 7 and 23 μm, could influence endothelial cell morphology and directional alignment to a certain extent. These structural features further impacted the biological behaviors of the endothelial cells [51]. Our study incorporated SFMG microgrooves with widths of 30, 60 or 90 μm and depths of 5 or 10 μm. Remarkably, the SFMG with a width of 90 μm and a depth of 10 μm demonstrated the highest capability in enhancing the proliferation, alignment and angiogenesis of endothelial cells. These results suggest that a width of 90 μm and a depth of 10 μm may be the ideal dimensions for microgrooves promoting angiogenesis.

Our findings revealed that both SF and SFMG coatings enhanced the proliferation, migration and angiogenesis of HUVEC and increased the blood vessel density in the peri-implant connective tissue *in vivo*. Notably, the SFMG group exhibited a more pronounced effect, suggesting that the enhanced angiogenic properties of SFMG coatings are attributed to the combined influences of SF bioactivity and the microgroove morphology, with the latter demonstrating a more pronounced regulatory impact on the cell behaviors of HUVECs.

Angiogenesis is a complex process that involves a coordinated interplay of multiple growth factors related to vascular formation [52]. VEGF-A and bFGF play crucial roles in promoting blood vessel formation [53], with VEGF-A primarily regulating endothelial progenitor cell recruitment, proliferation and differentiation through the VEGF-VEGFR pathway during vascular development [54]. Similarly, bFGF is an essential angiogenic regulator that not only supports vascular formation and stabilization but also works synergistically with VEGF-A to enhance wound healing processes [55]. Although our study has not conclusively established a direct correlation between the secretion of VEGF-A and bFGF by HUVECs and the specific dimensions of the microgrooves, we observed that variations in groove morphology significantly affect endothelial cell morphology and alignment. These changes were likely to influence the biological behaviors of the cells, including the secretion of key angiogenic factors. The physical cues provided by the microgroove dimensions were thought to offer mechanical signals that guide cell orientation and activate cellular processes essential for angiogenesis. Supporting literature suggested those specific topographies like microgrooves enhanced the adhesion, viability and expression of angiogenic factors in endothelial cells more effectively than uniform surfaces, thereby promoted angiogenesis [35]. This evidence underscored the importance of the interaction between endothelial cells and their microenvironment, facilitated by the unique physical features of the implant surface, in optimizing cellular responses.

Recent studies have demonstrated that mechanical stimulation from the extracellular environment, when transduced into the cell, triggers the reorganization of the cellular cytoskeleton, thereby influencing cellular behaviors [56]. Endothelial cells feature dynamic and adaptable elastic cytoskeletons, crucial for maintaining stable cell shapes and transmitting environmental mechanical signals [57]. Interaction of endothelial cells with surfaces exhibiting microgroove topography results in the mechanical stimulation of myosin II within the cell skeleton by the grooves, causing elongation and contraction deformations along the groove orientation [58]. The groove boundaries restrict the vertical growth of the cell skeleton, thereby regulating cell growth along the groove direction [59]. The endothelial cells of SFMG groups exhibited a well-organized pattern with elongated spindle-shaped cells aligned along the grooves, suggesting that the elongation of cellular cytoskeleton induced by SFMG coatings may be a crucial factor influencing endothelial cell behavior.

Furthermore, mechanical stimulation from the extracellular environment can induce changes in cellular metabolism. Glucose metabolism in endothelial cells plays a vital role in responding to mechanical stimuli and maintaining cellular functionality. Endothelial cells predominantly rely on glycolysis for energy production, with more than 85% of their energy generated through glycolytic processes [60]. During angiogenesis, glycolytic expression is upregulated to meet the increased bioenergetic demands of cell migration and proliferation. De Bock *et al.* [61] demonstrated that inhibiting the key glycolytic enzyme PFKFB3 led to a 40% reduction in glycolytic flux, significantly attenuating the angiogenesis of endothelial cells. This finding highlights the significant relationship between the metabolic preference for glycolysis in endothelial cells and their biological functions. In line with these findings, HUVECs cultured on SFMG-coated surfaces exhibited increased glycolytic activity and more pronounced angiogenic function. Inhibition of glycolysis in these HUVECs led to the suppression of angiogenic activities such as proliferation, migration, tube formation and VEGF-A expression, suggesting that the effects of SFMG coatings on HUVECs are dependent on glycolysis.

Various studies have demonstrated the crucial role of mechanotransduction in governing cellular behaviors in response to surface topography. However, the specific intracellular mechanisms underlying mechanical signal transduction warrant further exploration. Our research delved into the intracellular mechanism by which microgroove topography influences glucose metabolism in HUVECs. The RNA-sequencing results indicated the upregulation of the PI3K-AKT pathway in HUVECs of SFMG groups. The PI3K-AKT pathway is pivotal in mechanotransduction, impacting cell migration and proliferation by modulating cytoskeletal dynamics [62, 63]. Additionally, it serves as a crucial link between mechanical signals and cellular metabolism, acting as a key upstream signaling pathway in glycolysis [64]. Our study demonstrated the activation of the PI3K-AKT-mTOR signaling pathway in HUVECs, as indicated by the increased expression of PI3K and the phosphorylation levels of AKT and mTOR in the SFMG-coated group. Inhibition of PI3K negated the promoting effect of the microgroove coating on the expression of key genes involved in cellular glycolysis, confirming the pivotal role of PI3K-AKT pathway activation by SFMG coatings in promoting glycolysis and angiogenesis in HUVECs.

## Conclusion

In this study, the SFMG coatings on zirconia surfaces constructed by ink-jet printing exhibited a uniform and stable morphology. Microgrooves, measuring 90 µm in width and 10 µm in depth, effectively guided cell growth and enhanced the angiogenic abilities of HUVECs, including proliferation, migration and tube formation. Additionally, SFMG coatings regulated HUVECs by activating the intracellular PI3K-AKT pathway and downstream glycolysis. These findings introduce a novel approach to fostering angiogenesis in peri-implant connective tissues, offering significant insights into the surface design of zirconia abutments.
